# Quantification of the morphological characteristics of hESC colonies

**DOI:** 10.1038/s41598-019-53719-9

**Published:** 2019-11-26

**Authors:** Sirio Orozco-Fuentes, Irina Neganova, Laura E. Wadkin, Andrew W. Baggaley, Rafael A. Barrio, Majlinda Lako, Anvar Shukurov, Nicholas G. Parker

**Affiliations:** 1 School of Mathematics, Statistics and Physics, Newcastle University, Newcastle upon Tyne, NE1 7RU United Kingdom; 2 Institute of Genetic Medicine, Newcastle University, Newcastle upon Tyne, NE1 7RU United Kingdom; 3 Institute of Cytology, Russian Academy of Sciences, St-Petersburg, Russia; 4 Instituto de Física, Universidad Nacional Autónoma de México, Mexico City, Mexico

**Keywords:** Embryonic germ cells, Applied mathematics

## Abstract

The maintenance of the undifferentiated state in human embryonic stem cells (hESCs) is critical for further application in regenerative medicine, drug testing and studies of fundamental biology. Currently, the selection of the best quality cells and colonies for propagation is typically performed by eye, in terms of the displayed morphological features, such as prominent/abundant nucleoli and a colony with a tightly packed appearance and a well-defined edge. Using image analysis and computational tools, we precisely quantify these properties using phase-contrast images of hESC colonies of different sizes (0.1–1.1 ${{\bf{\text{mm}}}}^{{\bf{2}}}$) during days 2, 3 and 4 after plating. Our analyses reveal noticeable differences in their structure influenced directly by the colony area ${\boldsymbol{A}}$. Large colonies (*A* > 0.6 mm^2^) have cells with smaller nuclei and a short intercellular distance when compared with small colonies (*A* < 0.2 mm^2^). The gaps between the cells, which are present in small and medium sized colonies with *A* ≤ 0.6 mm^2^, disappear in large colonies (*A* > 0.6 mm^2^) due to the proliferation of the cells in the bulk. This increases the colony density and the number of nearest neighbours. We also detect the self-organisation of cells in the colonies where newly divided (smallest) cells cluster together in patches, separated from larger cells at the final stages of the cell cycle. This might influence directly cell-to-cell interactions and the community effects within the colonies since the segregation induced by size differences allows the interchange of neighbours as the cells proliferate and the colony grows. Our findings are relevant to efforts to determine the quality of hESC colonies and establish colony characteristics database.

## Introduction

Human embryonic stem cells (hESCs) are pluripotent cells, derived from the blastocyst-stage embryos, which have the capacity to differentiate and give rise to all tissues of the body. More than 20 years ago, a method to derive stem cells from human embryos was discovered and allowed the use of these cells for further research in vitro^1^. They provide an opportunity to study early human development and the processes by which the undifferentiated state is lost and differentiation into different tissues occurs^2^. The specific signalling factors promoting stem cells to remain unspecialised in culture without differentiation have been highly optimised during the last two decades^3^. Also, protocols have been developed to differentiate hESCs towards all three germ layers for disease modelling, cell-based therapies and drug screening^4^. After the derivation of human induced pluripotent stem cells (hiPSCs)^5^, which made creating patient-matched embryonic stem cell lines feasible, hESCs and hiPSCs have become an emerging model for developmental studies and personalised medicine^6–8^.

Although the genetic and signalling pathways that control pluripotency in hESCs have been described in the last decade^9–14^, much less is known about the factors that control the arrangement of the cells into a pluripotent colony and how this affects pluripotency. Human ESCs grow as a multicellular colony. At the single cell level the transcription factors (TFs) associated with the maintenance of pluripotency fluctuate stochastically^15,16^. These different expression states are maintained by different signalling, transcriptional, and epigenetic regulatory networks. However, pluripotency, considered as an emergent property of stem cell populations and their niches (rather than a property of single cells), is controlled by niche-mediated regulation in response to mechanical, chemical and physical stimuli^17,18^. Thus, understanding how pluripotency is affected by cell segregation within the bulk of a colony is of practical importance in generating and selecting the optimum clones, and automating this for industrial-scale production.

A quantitative analysis of the arrangement of the cells within the colonies is a prerequisite for the construction of hypothesis-driven mathematical and computational models that can provide explanations for the observed dynamics in hESC colonies and their regulation at the microenvironment level. To characterise how hESCs regulate their assembly into a multicellular colony we performed a detailed quantitative analysis of hESC colonies of different sizes during the exponential growth phase, at days 2, 3 and 4 after plating. These quantitative properties of the colony morphologies are poorly studied, while the pluripotent regulation at the expression level captures most of the attention^19–22^.

Previous works within our group have demonstrated that isolated hESCs growing on Matrigel^TM^with mTESR1 media are highly motile ($\sim 16.25\,\mu$m/h) and sensitive to the presence of nearby cells^23,24^.

The formation of hESC colonies in vitro is a natural process emerging when a single cell proliferates and forms a small cluster. The local density attained within the cultures should be highly relevant for the maintenance of the undifferentiated state. A recent study for human induced pluripotent stem cells (hiPSCs) has shown the migratory behaviours of the cells vary on different substrates (e.g., laminin, fibronectin, matrigel) due to changes in their adhesion properties, concluding that the regulation of the motility of the cells might improve the clonality of the forming colonies^25,26^. With the colony growth, the cells regulate each other through cell-cell and cell-media interactions^17,27^ resulting in community effects that regulate the undifferentiated and differentiated state^21^. Therefore, the results obtained at the single-cell level cannot be sufficient to deduce biological processes at the colony level as a whole.

In this work, we analyse colonies growing in feeder-free conditions (Matrigel^TM^). It has been demonstrated that cells within the same colony have a higher correlation of being of the same type, e.g. undifferentiated or primed towards differentiation^21^, which might be by the combination of endogenous signals between the cells and extrinsic factors (addition of differentiation cues).

The size and morphology of the colonies provide with preliminary information about the pluripotency status of the cells. The undifferentiated state is assessed through the specific morphology of the cells and the colonies, see Table 1, which is typically estimated visually. The morphological features of undifferentiated hESCs inside a colony are: roundness, large nucleus, scant cytoplasm and prominent (highly visible) nucleoli. As the colony grows, the central part becomes more compact than the periphery. Small hESC colonies show white spaces or gaps of medium compacticity between the cells^28–30^. To account for these features, we measured the individual cells by manually outlining their nucleus. This gives us the nuclei projected shape in the colony. Several properties, such as nucleus area and shape descriptors (aspect ratio, Feret’s diameter, circularity, roundness and solidity), defined in the supplementary material (SM)) were measured. Our results show that colonies with $A < 0.1\,{\text{mm}}^{2}$ show distinctive features in their structural properties, such as a large nucleus cell area and a large separation between nearest neighbours. Both quantities decrease as the colony size increases, with the largest colony showing the smallest value in the mean cell nucleus area. To measure the segregation of the small (recently divided) cells, we introduce a segregation order parameter. Our results suggest the self-organisation of the cells in terms of their nucleus sizes, since the small cells cluster together in patches, separating the larger cells from each other.

**Table 1:** Morphological features of hESCs and their colonies.

Type	Characteristics
Cell	Prominent nucleoli
	Scant cytoplasm
	Round
	Small
Colony	Round
	Flat
	Well-defined edges
	Gaps between the cells ($A < 0.6\,{\text{mm}}^{2}$)

Computational models are helpful to quantitatively analyse and improve the understanding of the processes that underlie fate decisions in hESCs and hiPSCs. However, before establishing the appropriate protocol for *in silico* approaches, it is important to quantify the morphological features frequently used in the visual identification of pluripotent hESC colonies, see Table 1, in agreement with previous publications^23,24,28–34^. These give us value information about the morphological properties of the cells arranged in colonies. In the future, this information will be integrated alongside other mechanisms that determine the behaviour of the system, to build algorithms of interaction rules aiming to understand their emergent properties^35^.

## Materials and Methods

### Cell culture and propagation

Human embryonic stem cells (hESCs) (H9 cell line, WiCell, Madison, WI) were passaged on 6-well plates coated with hESC-qualified Matrix at a 1:4 split ratio using an EDTA-based dissociation solution. 2 ml of mTERSR1 media was used per well. The cells were kept in small clumps avoiding the passaging of single cells (due to low rates of survival). We aimed to plate cell aggregates of approximately 15–20 cells each. The culture was kept for 4 days at $37$°C with a humidified $5{\rm{ \% }}{\text{CO}}_{2}$ atmosphere. The colonies were imaged at day 2, 3 and 4 after plating before they reached a $60\  \%$ confluency across the well.

**Figure 1:**
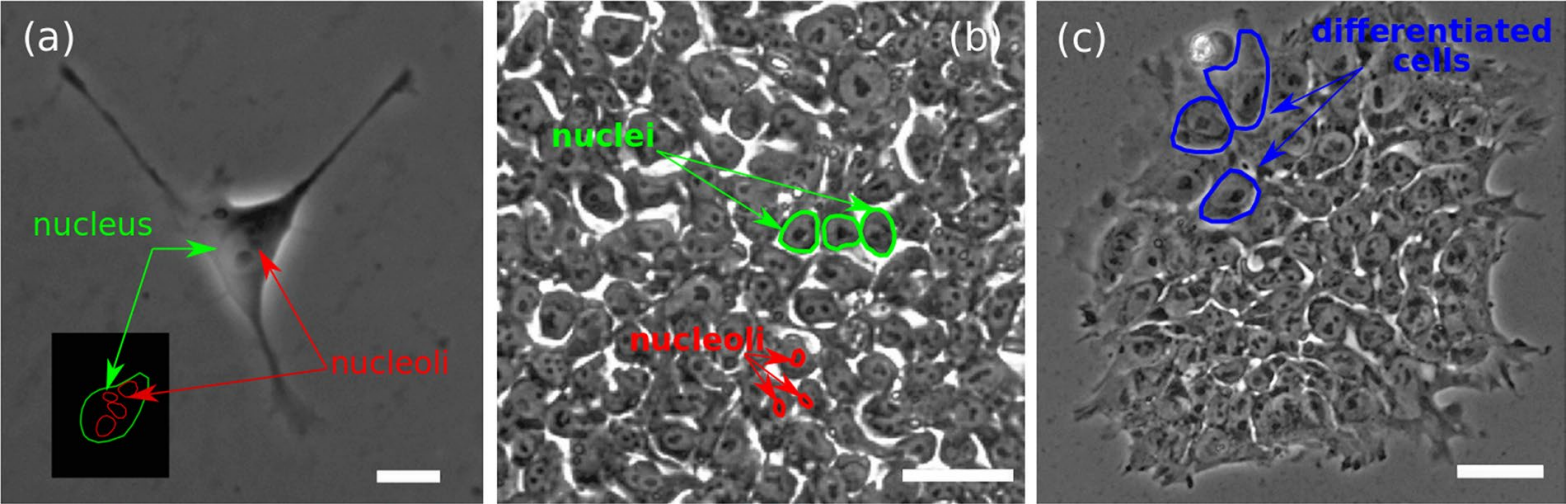
(**a**) Phase-contrast image of a single isolated hESC at day 2 after plating, showing a well-defined nucleus, nucleoli (black dots) and spreading lamellipodia. Bar $20\,\mu$m. (**b**) Detail of the spatial arrangement of cells within a colony, with well-defined nuclei and prominent nucleoli (black dots). The very distinctive gaps between the cells occur in colonies with areas $A < 0.6\,{\text{mm}}^{2}$. Bar $50\ \mu$m. (**c**) HESC colony in which the cells located at the top-left (outlined in blue) with larger nuclei. Bar $100\ \mu$m.

**Figure 2:**
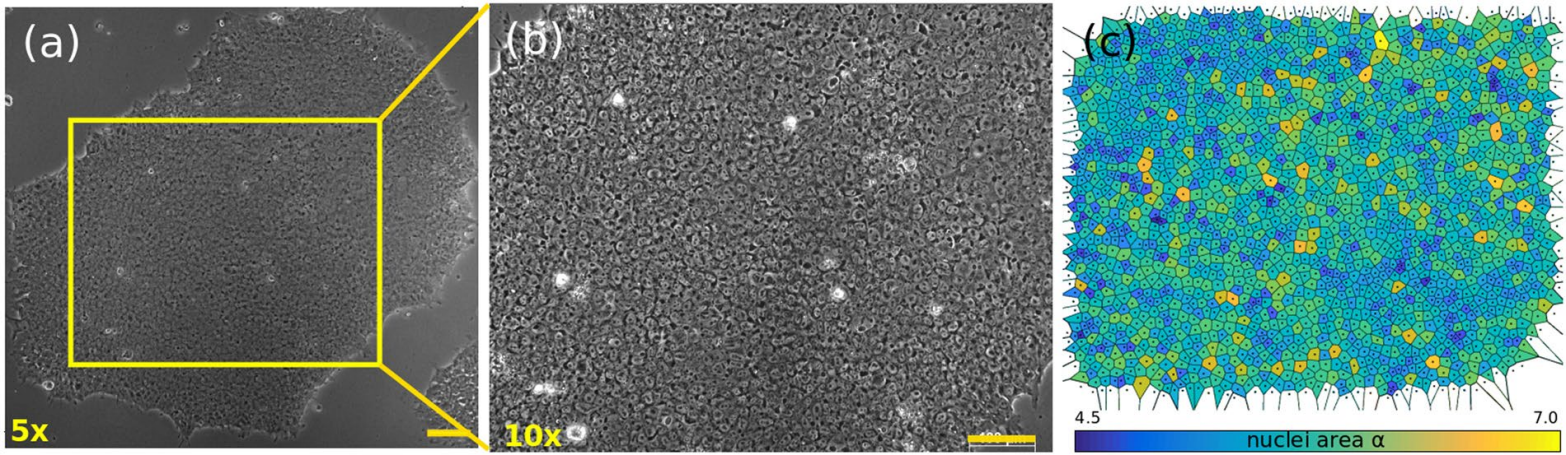
Phase-contrast images of a hESC colony at day 3 after plating. For large colonies we used (**a**) low magnification (5$\times$) to capture the boundaries and (**b**) higher magnification (10$\times$) to measure the cells features (the enclosed region in (**a**)). This colony is a densely packed example with no gaps within the cells. Bars $100\ \mu$m. (**c**) Voronoi tessellation obtained from the centroid position of the cells. The nuclei area is shown in logarithmic scale.

The ability of hESCs cells to form colonies depends on the cytoskeleton rearrangement, contraction of actin filaments, the interaction between the cells, and the timely function of regulatory proteins^36^. When isolated, the cells have their cytoskeleton and lamellipodia unfolded and spreading over the substrate, see Fig. 1(a). In colonies, the cells are close to each other as shown in Fig. 1(b). This section of a colony contains several cells in which the nuclei, nucleoli (dark spots) and gaps (white spaces between the cells) are easily detected. Larger and denser colonies do not show gaps and the cells are closer to each other, see Fig. 2.

### Phase-contrast microscopy

We studied the colonies using phase contrast microscopy, since this method allows the cells to behave as naturally as possible without the need to stain the cells with fluorescent dyes which may induce photo-toxicity^37^ and possible changes in cell behaviour^38^. The image acquisition was performed in an inverted microscope Axiovert 200 M with Zeiss AxioCam HRc (Carl Zeiss^TM^) using two objective magnifications 5$\times$ (Plan Neofluar Ph1 5$\times$/0.15) and 10$\times$ (Plan Apochromat Ph1 10$\times$/0.32), with the following scalings 1.36 $\mu$m/pixel and 0.67 $\mu$m/pixel, respectively. Images are saved in TIFF format (size 1300 $\times$ 1030 pixels) with no additional compression, see Supplementary Fig. S11. The imaging was performed every 24 h at multiple x-y locations per well to obtain an adequate sample of the cells for 3 days until the confluency of the cells was about $60\  \%$, meaning that most of the colonies did not merge with each other and were quasi-bidimensional structures.

Examples images of hESC and their colonies are shown in Fig. 1. The internal structure of a single isolated cell is shown in Fig. 1(a) with a scheme at the bottom-left side outlining the nucleus and nucleoli. Colonies of varying size were selected with morphological features typical of undifferentiated colonies, i.e., with clear borders, containing small round cells with large nuclei and notable nucleoli^28^; an example is shown in Fig. 1(b). Figure 1(c) shows a hESC colony with cells showing a different nucleus/cytoplasm ratio.

To quantify the morphological characteristics within the colony we outlined each nucleus manually and extracted several parameters such as the centroid position, nucleus area and relevant shape descriptors included in ImageJ^39^. During mitosis, the cells adopt a spherical shape, detach from the ECM, divide and reattach again, with the two new daughter cells lying in close proximity to each other. We recorded these mitotic events during the manual tracing of the cell nuclei.

We outlined the nuclei of the cells in $19$ colonies of different sizes (see Supplementary Table S2 in the SM for further details). Alongside this information, the boundaries of 38 colonies were obtained using an edge detection algorithm through a canny Deriche filtering^39^, see Supplementary Table S4 in the SM for more details. An example of the analysis performed on the colonies is shown in Fig. 2(a). This sample has an area $A=1.132\,{\text{mm}}^{2}$ and it was imaged at day 3 after plating. For large colonies, we imaged the structure at low magnification (5$\times$) to account for the colony’s features, and at a higher magnification (10$\times$) focusing in the bulk, Fig. 2(b), to outline the cell nuclei, Fig. 2(c). Using ImageJ^39^ software (http://rsb.info.nih.gov/ij/), we processed the outlined images and obtained the centroid position, area, perimeter and shape descriptors (aspect ratio, solidity, circularity and Feret’s diameter) of each nucleus and, at a larger scale, of each colony.

### Voronoi diagram

The spatial data analyses presented in this work are based on the Voronoi diagram (VD) that divides the area in the most equalitarian fashion, in such a way that the area occupied by a cell is obtained by tracing straight lines between the position of a cell and all its neighbours and drawing a perpendicular line in the middle. These perpendicular lines form a convex polyhedron, called the Voronoi cell. Therefore the VD is the collection of Voronoi cells. The generated “cells” are not uniform in shape and their number of faces vary from one to another.

The geometric dual of the VD is called the Delaunay triangulation (DT). It connects those points of a VD that share a common border. The VD facilitates spatial analysis, e.g., the closest neighbours identification through the adjacency matrix, and is used in many fields of science, including cell biology^40,41^. We used the VD to measure the structural properties of the colonies and the DT to obtain the intercellular distances.

**Figure 3:**
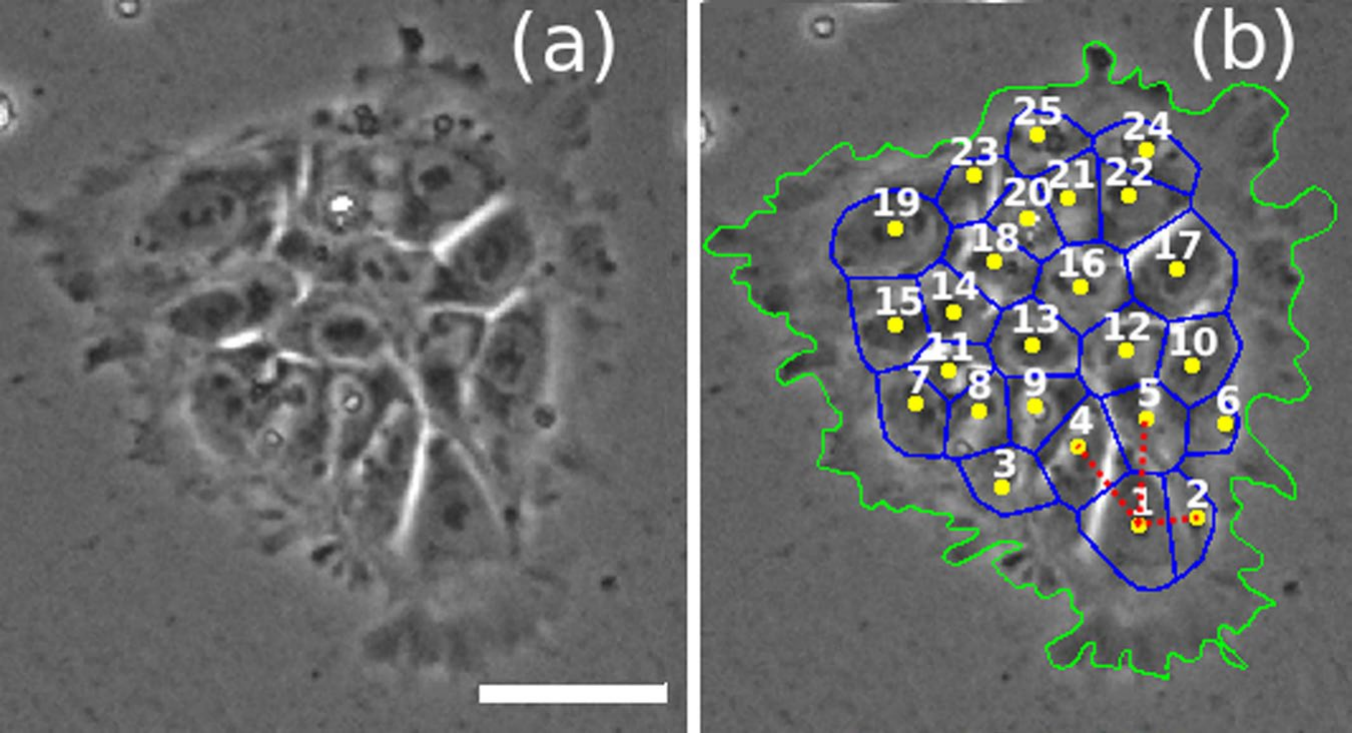
(**a**) The VD for a small colony with 25 cells and (**b**) constructed through the set of centroid positions of the cells. The dotted (red) lines show the first nearest neighbours for cell 1. The green line is the outline of the colony border. Scale bar: $50\ \mu$m.

As an example, Fig. 3 shows the VD for a small colony with 25 cells. The nearest neighbours of cell 1 are connected with dotted (red) lines (DT), i.e., cells 2, 4 and 5, giving the distance to the nearest neighbours. The cells 3 and 6 are the second nearest neighbours of cell 1. We performed this analysis on larger colonies, see Fig. 2(a). An example of the Voronoi tessellation obtained for the region in Fig. 2(b) is shown in Fig. 2(c) for 1982 cells. The nuclei are coloured according to the logarithm of the nucleus area ($\alpha$) to ease in the visualisation (see colorbar). Using the centroids as input points, we obtained the VD, shown with black continuous lines. Since the cells are round in shape (see SM, Supplementary Fig. S2, the VD allows accurate identification of each cell’s neighbourhood. Through the DT, we identified the nearest neighbours associated to each cell and calculated the mean number of nearest neighbours $\langle {N}_{n}\rangle$ and the mean distance to nearest neighbours (or intracellular distance) $\langle {\ell }_{n}\rangle$, measured from the centroid position of the two cell pairs. From now on, the angular brackets $\langle \rangle$ will denote the average taken over the cell population within a given colony. The bar $-$ will denote the average taken over several colonies.

## Results

After plating, hESCs form small clusters of several cells attached to each other and to the ECM. These are the initial seeds from which larger colonies start to grow through proliferation. The cells inside the colonies display self-propulsion, resulting in a movement of the colony as a whole through the culture.

Depending on the initial plating density, merging of colonies might occur after some time. In our experiments, the confluency of the colonies was less than 60% on day 4. Due to the variability in single cell movements, cell growth and mitotic events, we hyphotesize that the biophysical interactions between the cells in the colonies are not distributed uniformly. As a result, the colonies become more irregularly shaped when the number of cells increases.

### Single hESCs areas

**Figure 4:**
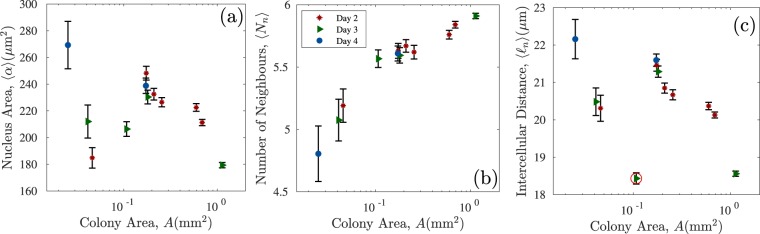
Mean values of the (**a**) nucleus area $\langle \alpha \rangle$, (**b**) the number of nearest neighbours $\langle {N}_{n}\rangle$, and (**c**) the intercellular distance $\langle {\ell }_{n}\rangle$ as a function of the colony area $A$. The standard error is shown as a black line around the data points. Data points are presented with different symbols according to the day at which the image was taken (see legend in panel (b)).

The mean nucleus area $\langle \alpha \rangle$, mean number of nearest neighbours $\langle {N}_{n}\rangle$ and mean intercellular distance $\langle {\ell }_{n}\rangle$ of the colonies are shown in Fig. 4 as a function of colony area $A$. The mean cell nucleus area $\langle \alpha \rangle$, Fig. 4(a), shows high variability between colonies of different sizes and sampling days. The smallest colonies, with $\sim 70$ cells at day 3 () and 25–46 cells at day 4 (), have the largest mean nucleus area, with $\langle \alpha \rangle =269\pm 18\ (\pm 111)\ \mu$m${}^{2}$ and $212\pm 12\ (\pm 104)\ \mu$m${}^{2}$ respectively. The small colonies analysed at day 2 with $\sim 115$ cells () result in $\langle \alpha \rangle =184\pm 8\ (\pm 82)\ \mu$m${}^{2}$, a lower value than their later imaged counterparts. The quantities shown immediatly after the measurement are the standard errors of the mean measured through the relation $\sigma /\sqrt{{N}_{c}}$, where $\sigma$ is the sample standard deviation and ${N}_{c}$ is the number of cells in the colony (number of observations in the sample). Alongside this, we show inside the parenthesis the standard deviation around the mean. The standard deviations are large since we included cells undergoing mitosis, this result indicates that the cell nucleus is larger in the small colonies formed at later stages of passage, *e.g* day 4 colonies with a few dozen cells.

The mean number of nearest neighbours $\langle {N}_{n}\rangle =4.8\pm 0.22(\pm 1.4)$ cells (day 4), $5.1\pm 0.17(\pm 1.4)$cells (day 3) and $5.2\pm 0.13(\pm 1.4)$ cells (day 2) increases with the colony area $A$, whilst the mean intracellular distance $\langle {\ell }_{N}\rangle$ shows the same trend as $\langle \alpha \rangle$ as a function of $A$. For small colonies at day 4 with large cell nucleus areas, our results show cell-to-cell contacts between approximately five cells that have a larger separation between them.

For colonies containing between 350 and 550 cells, we obtain $\overline{\langle \alpha \rangle }=239\pm 5(\pm 104)\ \mu$m${}^{2}$. The nucleus area is between 220–240 $\mu {\text{m}}^{2}$ and shows only modest variability between days 2 and 3. The mean number of neighbours $\langle {N}_{n}\rangle$ increases steadily with the colony area, Fig. 4(b), whilst the mean intercellular distance $\langle {\ell }_{n}\rangle$ decreases, i.e. the colony becomes more compact, Fig. 4(c).

The day 3 colony highlighted with the red circle in Fig. 4(c) has a particularly short intracellular distance for its area. The image of this colony is shown in the Supplementary Fig. S9(a), we observe patches of small cells throughout the colony which can be easily detected through the Voronoi tessellation, see Supplementary Fig. S9(b); this might indicate the interplay of other factors in the rearrangement of the cells in this sample this might indicate the interplay of other factors in the rearrangement of the cells in this sample, possibly due to the colony being very young and un-established. Although the mean cell area for this colony is around 200 $\mu {\text{m}}^{2}$, this sole measurement would not be sufficient to set this colony apart from the others. Therefore, the Voronoi tessellation applied to measure the intercellular distances is giving extra information on the cell arrangement within the colony.

Finally, for the largest colonies analysed (the last three points to the right in Fig. 4), there is a clear decrease in the mean nucleus area with colony size. The largest colony has the highest mean number of neighbours and the smallest inter-cell distances. Therefore, for large colonies (higher density) on average six neighbours are involved in cell-to-cell interactions. Visually these colonies are very dense, cells are tightly packed and there are no gaps between them.

In summary, colonies with $A < 0.1{\text{mm}}^{2}$ and ${N}_{c} < 100$ cells have the largest nuclei and intercellular distances with less neighbouring cells. On the other hand, the largest colony has cells with the smallest nuclei, short intercellular distances and six neighbours on average. Since the number of nearest neighbours increases with the colony area (and the number of cells) we suggest that the number of cells in a colony increases faster than its area as the cells fill the space within the colony.

We measured several other properties for the nuclei, such as their aspect ratio, perimeter, Feret’s diameter, circularity, roundness and solidity, as shown in the Supplementary Figs. S2 and S3 in the SM. Some of these parameters have been used to characterise mouse embryonic stem cell (mESCs) colonies during differentiation^42^. However, for hESCs, at the single and colony level, these measurements do not show any significant change in behaviour that would indicate changes in the the morphology of the cells and colonies in terms of the days after plating and colony sizes, see Supplementary Tables S3 and S4.

### Probability distribution functions of nuclei area

**Figure 5:**
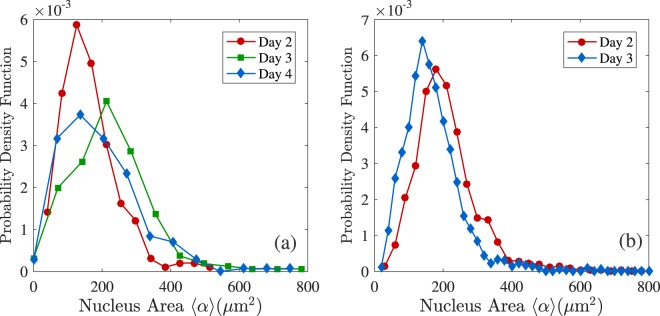
Probability density function (PDF) for the nucleus area ($\alpha$) measured for (**a**) colonies imaged at day 2, 3 and 4, with an area $A < 0.1{\text{mm}}^{2}$ and (**b**) the largest colonies imaged at day 2 and 3.

The size and shape of the cells are good indicators of their health and most importantly of their viability as a pluripotent cell for stem cell research. The averages of the quantities obtained in the previous section give a rough estimation of the behaviour of these variables in terms of the colony sizes. However, to account for the variability of the nuclei areas within a colony, we calculated the probability distribution functions PDF of $\alpha$, shown in Fig. 5, for several samples, dividing them according to sampling day and size.

Colonies with $A < 0.2\,{\text{mm}}^{2}$ (day 4) show an abrupt change in $\langle \alpha \rangle$ as a function of sampling day. Both day 3 and 4 colonies have a broader distribution, with cells having nuclei of sizes $\alpha  > 600\ \mu$m${}^{2}$. The colonies at day 2 have a narrower distribution in which all nuclei have an area $\alpha  < 500\ \mu$m${}^{2}$. It is important to keep in mind that day 3 and 4 colonies have half as many cells as the day 2 colonies, see Supplementary Table S2 in the SM for further details. Overall, we observed a similar PDF for the smallest colonies, which becomes narrower as the cells increase their numbers. Therefore, a large nucleus in small samples may be due to a lack of compactness and pressure between the cells.

For the largest colonies analysed at days 2 and 3, with $\langle {N}_{c}\rangle$ = 1257 $\pm$ 327 and 1982 cells, respectively, the distributions become narrower as the colonies get bigger, see Fig. 5(b). Occurrences of nuclei area $\sim 350\mu {\text{m}}^{2}$ disappear, and overall the cells become more homogeneous in size with $\langle \alpha \rangle =179\pm 87\mu {\text{m}}^{2}$ for the day 3 colonies, see Fig. 2. Under these circumstances, crowding effects due to mechanical cell competitions may take place in the bulk.

### Colonies properties

During colony formation, there are physical forces transmitted through the cells that affect the local mechanical properties and, therefore, play important roles in cellular behaviour such as adhesion properties, cell proliferation, differentiation and death (through the activation of biochemical signals)^36,43–45^. The colony shape is one of the qualitative features used to identify the best colonies and best clones. To quantify their form, we obtained the area $A$, perimeter $P$ and shape descriptors of 38 colonies, see Supplementary Table S4 in the SM. To measure changes in cell and colony morphologies as the cell numbers increase, we counted the cells in 19 colonies and added these results to the other 19 colonies analysed in the previous section.

**Figure 6:**
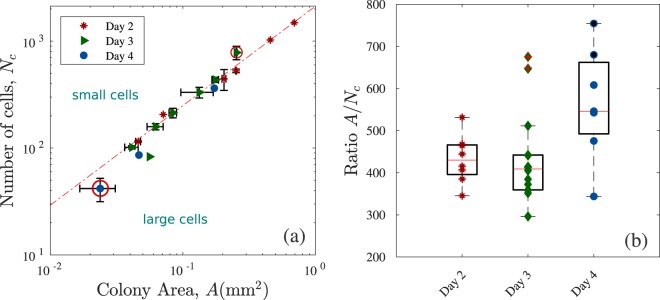
(**a**) The number of cells ${N}_{c}$ as a function of the colony area $A$ (log-log scale). Data points are coloured according to the number of days after plating in which the image was taken. The red dotted line corresponds to the best fit to ${N}_{C}(A)=\kappa {A}^{\beta }$, with a scaling factor $\kappa =2130$ and exponent $\beta =0.93$ (${R}^{2}$ = 0.97). The three outliers for $A < 0.01{\text{mm}}^{2}$ correspond to colonies with distinctive features when compared to the rest (partly differentiated). (**b**) Mean area per single cell, $\bar{A}=A/{N}_{c}$ are the following $\langle A/{N}_{c}\rangle =433\pm 57\ \mu$m${}^{2}$ (day 2), $434\pm 109\ \mu$m${}^{2}$ (day 3) and $564\pm 135\ \mu$m${}^{2}$ (day 4). The medians are shown as red central lines on each box, which edges represent the 25th and 75th percentiles respectively. Some data points at day 3 (crossed on top with +) were considered outliers. The points that correspond to colonies with differentiated cells are filled in black.

Figure 6(a) shows the the number of cells ${N}_{c}$ as a function of colony area $A$. A power function trend line, ${N}_{c}=\kappa {A}^{\beta }$, is appropriate with scaling factor $\kappa =2130$ and exponent $\beta =0.93$ (${R}^{2}$ = 0.97), see red dotted line. The exponent $\beta$ is approximately one, which corresponds to the cells maintaining the same nucleus area while the colony grows. The two largest colonies at day 2, with $A=0.456{\text{mm}}^{2}$ and $0.691{\text{mm}}^{2}$, follow the same trend. However, small colonies from day 4 (left red circle) and some samples on day 3 (right red circle) deviate from this relationship. We show both horizontal and vertical error bars for some data points to indicate measurements performed on more than one colony.

We detect five small colonies, imaged at day 4 (with ${N}_{c} < 100$), whose overall behaviour indicated exceedingly large nuclei, see Fig. 1(c) and Supplementary Fig. S9(d) in the SM. Those colonies are highlighted in Fig. 6(b) with black points and are detected as outliers by the boxplot method. A more detailed analysis of these colonies indicates that a proportion of the cell population has undergone differentiation, see Fig. 1(c).

Comparing colonies of similar sizes measured at day 2, $$A=0.252\pm 0.002{\text{mm}}^{2}$$, and day 3, $$A=0.254\pm $$$$0.003\,{\text{mm}}^{2}$$, we observe that the former has fewer cells ($${N}_{c}$$ = $$528\pm 20$$) than the latter ($${N}_{c}=782\pm 112$$). Therefore, there is an increment of $$\Delta {N}_{c}\approx 250$$ cells in the bulk of the colony without an increase in the colony area. We can infer that the disappearance of the gaps between the cells, highly visible at day 2, is a result of newly dividing cells filling the voids. Consequently, the power-law relationship (linear on log-log scale) shown in Fig. 6(a) between $$A$$ and $${N}_{c}$$ holds only for colonies at day 2 and some colonies on day 3.

We observe that small colonies ($A < 0.3\,{\text{mm}}^{2}$) show gaps between the cells. For larger colonies, these gaps start to disappear in the middle of the structure and are completely lost in the largest colonies ($A > 0.6{\text{mm}}^{2}$). It is known that hiPSC colonies form actin (a linear polymeric micro-filament) fences encircling the colony that exerts extensive mechanical stress to enforce colony morphology and compaction^46^. We suppose that at initial stages of colony formation the cells accommodate themselves in such way that they have a higher intercellular distance between them, without being tightly packed^29,30^ forming a polymeric fence around them to enforce compactness^46^. For small and medium-sized colonies with gaps, there should be an outward pressure flow of cells at the boundary in order to accommodate newly divided cells in the bulk while keeping these spaces empty^47^.

With the increase in cell numbers, we assume that there are more mitotic events in the colony and less time to re-organise the colony edges. Therefore it is possible that the fences formed at previous stages continue to maintain a strong adhesion at the border with the ECM, making the filling of gaps possible.

### Segregation and population mixing

Segregation of cells in tissues and during pattern formation is an important phenomenon that occurs during the early phase of embryonic development, which ends with the formation of the three germ layers^48^. The arrangement of cells in the embryo occurs due to changes in the environment (surface cues) that induce differences in adhesion properties and changes in the cytoskeleton^49^. These differences in adhesion properties between neighbouring cells maintain a physical separation between different cell types, and it is one of the basic mechanisms for the pattern formation during development and wound healing^50^. Although *in vivo*, migration of hESCs is responsible for the segregation into physically distinct regions after a few rounds of divisions, *in vitro*, the presence of migratory effects is undesirable due to population mixing and loss of clonality^26^.

From experiments on isolated hESCs, our measurements indicate that the cell grows until it reaches a size of $\sim 300\ \mu$m${}^{2}$ (unpublished results), after which it divides into two almost identical cells of sizes $150\ \mu$m${}^{2}$. To account for size segregation in hESC colonies, we explore if the small cells are located away from larger cells in the colony.

To measure if the small cells are segregated from the largest cells in the bulk of the colony, we introduce a segregation order parameter depending on the level of separation between small (type A) and large (type B) cells. Several order parameters can be introduced to characterise processes of segregation according to several segregation criteria^51^.

The VD, see Section §2.3, identifies accurately the number of nearest neighbours in each colony. We introduce a suitable segregation order parameter that depends explicitly on the number of nearest neighbours. We consider two types of particles A and B, if the system is segregated, each particle A will have more neighbours of the same type. The segregation order parameter $\delta$ is defined as follows,

1 $$\delta =1-\frac{{N}_{c}{N}_{AB}}{{N}_{n}{N}_{A}{N}_{B}},$$

where ${N}_{AB}$ is the sum of the number of A Delaunay neighbours that B particles have, double counting the A particles that are neighbours of different B particles and ${N}_{n}$ is the number of nearest neighbours that each particle has on average. For a perfectly mixed system, with ${N}_{n}=6$ Delaunay neighbours, equation 1 results in $\delta \approx 0$. If the system is completely segregated, for example, one cluster of A particles surrounded by other of B particles, $\delta  \sim 1$. The calculation of $\delta$ was performed for the largest colonies analysed each day for which the mean number of nearest neighbours exceeded $\langle {N}_{n}\rangle  > 5$, see Fig. 4(b).

**Figure 7:**
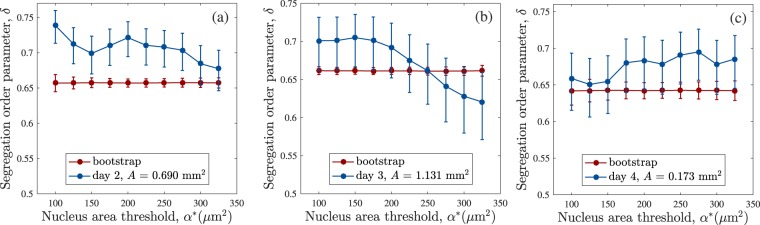
Segregation order parameter $\delta$ for the colonies with areas (**a**) $A=0.690\,{\text{mm}}^{2}$ (day 2), (**b**) $A=1.131\,{\text{mm}}^{2}$ (day 3) and (**c**) $A=0.173{\text{mm}}^{2}$ (day 4). The segregation is calculated in terms of an area threshold ${\alpha }^{\ast }$ as a proxy for two cell types. Type A cells ($\alpha  < {\alpha }^{\ast }=200\ \mu$m${}^{2}$) are segregated from the larger type B cells in (**a**,**b**). The results are inconclusive for c. The values of $\delta$ obtained by re-sampling the data sets (bootstrap method) are shown alongside these results, see the legend in the inset.

We use an area threshold ${\alpha }^{\ast }$ to group the cells into two categories: type $A$ cells ($\alpha  < {\alpha }^{\ast }$) and type B cells ($\alpha \ge {\alpha }^{\ast }$) and vary ${\alpha }^{\ast }$ between $100\ \mu$m${}^{2}$ and $325\ \mu$m${}^{2}$. Applying equation 1 to the largest colonies measured each day, gives the results shown in Fig. 7. We also show the results of the bootstrap to detect differences with a re-sampled data set. The colonies with areas $0.690{\text{mm}}^{2}$ (day 2) and $1.131\,{\text{mm}}^{2}$ (day 3) contain ${N}_{c}=1489$ and $1982$ cells respectively, panel a and b, whereas the colony with area $0.173{\text{mm}}^{2}$ have ${N}_{c}=363$ cells, panel c. Our results strongly suggest that, in large colonies, small cells ($\alpha  < 200\,\mu {\text{m}}^{2}$) are segregated from larger ones. In both cases, see panels a and b in Fig. 7, the curve for $\delta$ is above the one obtained from the bootstrap method. For ${\alpha }^{\ast } > 275\ \mu$m${}^{2}$, in Fig. 7(b), $\delta$ reaches lower values that the bootstrap; this means that cells with $\alpha  > 275\ \mu$m${}^{2}$ have less chance of having neighbours of the same size, and therefore, they are surrounded by smaller particles which are clustered together. As we increase ${\alpha }^{\ast }$ beyond $200\,\mu {\text{m}}^{2}$, $\delta$ decreases reaching the values for the random configuration. Therefore, for the largest colony, Fig. 7(b), the cells are separated in a random fashion at ${\alpha }^{\ast }$ = $250\,\mu {\text{m}}^{2}$. However, for ${\alpha }^{\ast }$ > $300\,\mu {\text{m}}^{2}$, $\delta$ continues decreasing until it reaches $0.66$, which indicates that the larger cells within the colony (mitotic events) tend occur far apart from each other. The results shown in Fig. 7(c), for the colony with $A=0.173\,{\text{mm}}^{2}$ remain within the values obtained with the bootstrap method and the results are inconclusive, suggesting the need of larger colonies to obtain accurate measurements.

## Discussion

We quantified the morphological and structural properties as well as the behaviour of hESCs during colony formation using phase-contrast images. Human embryonic stem cells self-organise into colonies with sharp edges and a strong adhesion at the border that promotes the maintenance of the pluripotent state by keeping the colonies tightly packed^14,46,52^. Our analyses reveal that the colonies change their morphological properties as the cells proliferate and the structure becomes larger.

We derived the relationships between the cells nuclear areas, number of neighbours and intercellular distance as a function of colony areas and days after plating. These results show a high variability for both cell areas and intercellular distances between samples. From these measurements, we conclude that a characteristic cell area can not be defined in terms of the size of the colony or stages of experimentation. Difficulties in measuring the properties of hESC colonies have been reported elsewhere, see for example^29,30^. Particularly in^30^, the authors report an average size for a hESC (H9 cell line) in a “mature colony”, measured using phase-contrast images: $\langle {\alpha }^{\dagger }\rangle \sim 315\,\mu {\text{m}}^{2}$. In this work we obtained $\langle \alpha \rangle \sim 180\,\mu {\text{m}}^{2}$ for a colony with area $A=1.1\,{\text{mm}}^{2}$. This variability in the averages of the morphological properties of hESC highlight the difficulties in the development of automatic detection algorithms capable of discerning healthy/bad colonies in terms of these properties.

The number of cells follows almost a linear relationship with the colony areas for $A < 0.8\,{\text{mm}}^{2}$, which agrees with previous results presented by^30^. We expect that larger colonies will not follow a linear relationship, since as the colonies get more dense the cells get smaller, e.g. $\langle \alpha \rangle \sim 180\,\mu {\text{m}}^{2}$ for $A=1.1\,{\text{mm}}^{2}$. The number of first nearest neighbours available for each cell increases for larger colonies. For example, each cell within the largest colony, interacts, by contact, on average with six other cells.

The segregation of the cells is measured through a segregation order parameter. Our results suggest the self-organisation of the cells in terms of their nucleus sizes, since the small cells cluster together in patches, separated from larger cells. Recent results by^21^, using micropatterned colonies of a few cells, indicate that interactions between neighbours can lead to sustained and homogeneous signalling for differentiation. In large colonies, the emergence of collective effects must be at play which results in the smallest individuals to cluster in patches within the colony. Since the analysed colonies were grown on Matrigel^TM^, their migratory effects are large^23,24^ and this could be a relevant factor in the spatial organisation of the cells within the colony. The continuous re-organisation of the colonies implies that neighbours are interchanged continually and consequently the cell population is continuously mixed; this directly influences the level of clonality within the colonies and the outcome of community effects that will furthermore influence the pluripotency achieved by the population^21,26^.

Recent studies on hiPSCs with modified molecular regulators of cortical tension and cell-cell adhesion (through target genes ROCK1 and CDH1, respectively) have shown the emergence of distinct patterning events within hiPSC colonies through cell-driven segregation that dictated the colony organisation without the loss of pluripotency^53^. Our results indicate that newly divided (small) cells are driven away from larger cells, clumped together in patches. Whether this effect is solely due to the mechanical effects (pushing) between the cells or changes in the cells’ cortical tension/adhesion properties along the cell cycle remains unknown and its elucidation requires further work.

## Conclusion

The morphological analysis of hESC colonies is a powerful non-invasive tool to evaluate their quality and choose the best clones for medical applications, unlike invasive labelling procedures that involve genetic manipulation. Although the implementation of an algorithm for the automatic detection of cells within a colony was beyond the scope of this work, once such a method is developed, the parameters estimated throughout this paper can be easily implemented at a larger scale, to quantify accurately the parametric properties of pluripotent colonies.

Our work indicates that the mean nuclei area and mean distance between nearest neighbours might be good parameters to detect changes in the morphology of the colonies, despite the inherent variability in the cell sizes associated to the cell growth and the cell cycle. Our algorithms detect that small colonies at day 4 show distinctively larger cell nuclei and intercellular distances. These changes in their morphological properties might affect their pluripotency levels. Assuming an average hESC cycle duration of 14.6 h^22^ and an exponential colony growth starting from a single founder cell, we estimate that day 4 colonies with $N=[25,46]$ cells, were formed between 2–3 days before imaging. Therefore, later formed colonies have cells with changed morphological characteristics. Following this same premise, the larger colonies analysed at day 2 and 4 with thousands of cells, most certainly did not started from single founder cells. Our results suggests that this might be advantageous for the maintenance of their structural properties. The segregation of the cells inside the colony has strong biological implications in regards of the genetic and phenotypic spreading, since neighbouring individuals eventually end up in completely different locations.

## Supplementary information

Supplementary Material

## Data Availability

The datasets used and/or analysed during the current study are available from the corresponding author on reasonable request.
