# Supplementary material for: Quantification of the morphological characteristics of hESC colonies
Source: Sci Rep. 2019 Nov 26;9:17569. doi: 10.1038/s41598-019-53719-9 (PMC6879623; doi:10.1038/s41598-019-53719-9)
Supplement: Supplementary file 1 — Supplementary Material [file 41598_2019_53719_MOESM1_ESM.pdf]

Supplementary Material: Quantification of the morphological characteristics of hESC colonies

Sirio Orozco-Fuentes[1,*], Irina Neganova[2,3], Laura E. Wadkin[1], Andrew W. Baggaley[1], Rafael A. Barrio[4], Majlinda Lako[2], Anvar Shukurov[1], and Nicholas G. Parker[1]

[1] School of Mathematics, Statistics and Physics, Newcastle University, NE1 7RU, United Kingdom.
[2] Institute of Genetic Medicine, Newcastle University, NE1 7RU, United Kingdom.
[3] Institute of Cytology, Russian Academy of Sciences, St-Petersburg, Russia.
[4] Instituto de Física, Universidad Nacional Autónoma de México, Mexico City, Mexico.
[*] sirio.orozco-fuentes@newcastle.ac.uk

Nuclei detection

The analysis of each colony was performed using ImageJ [39] and the statistical analysis performed with Matlab®2016b. Each cell was manually traced and processed using ImageJ as it is shown in the Supplementary Figure S1. We obtained the nuclei area α and perimeter p, alongside the properties that we describe in the following.

Aspect Ratio

The aspect ratio η_{AR} is the relationship between the height and width of the nucleus. For a perfect circle, we obtain $\eta_{\mathrm{AR}} = 1$, elongated particles have $\eta_{\mathrm{AR}} > 1$. It is measured following the equation, $\eta_{\mathrm{AR}} = \frac{a}{b}$, where a is the major axis and b the minor axis of a rectangle that encloses the nucleus, in terms of the minimum area of enclosure.

The Supplementary Figure S2(a) shows the PDF for the nuclei aspect ratio η_{AR} following the colonies sampling day. Overall, the majority of cells have an aspect ratio of $\eta_{\mathrm{AR}}<2$. Cells with elongated shapes $\eta_{\mathrm{AR}} > 3$ are very few. This supports the applicability of the VD for an accurate tessellation of the colony.

Perimeter

The probability density function for nuclei perimeter is shown in Supplementary Figure S2(b). It has a mean of $\sim 50\,\mu$m, and this value is similar for all sampling days.

Feret's Diameter

The Feret's diameter (η_{Feret}), also know as maximum caliper measures the longest distance between any two points along the nucleus boundary. The probability density function (PDF) for the Feret's diameter for the nuclei are shown in the Supplementary Figure S2(c), according to the sampling day. The PDFs have a mean $\sim 21\,\mu$m, with the maximum caliper being $\sim 50\,\mu$m.

Circularity

Circularity is a shape descriptor that indicates the degree of similarity with a circle, therefore as this quantity approaches 0, the shape is less circular. It is calculated using the equation $\eta_{\phi} = \frac{4\pi\alpha}{p^2}$, with α and p the nucleus area and perimeter respectively. The values for the PDF of the circularity are shown in the Supplementary Figure S2(d), with all three distributions centred around ~ 0.85, therefore the nuclei shapes are highly circular.

Roundness

It is very similar to circularity but is insensitive to irregular borders along the perimeter. It is measured using the highest axis of the best fit ellipse. The equation for the roundness is $\eta_{\mathscr{R}} = \frac{4\alpha}{\pi a^2}$. The results for the PDF for the roundness are shown in the Supplementary Figure S2(e).

Solidity

Describes the extent to which a shape is concave or convex. The solidity of a completely convex shape is 1, the farther the solidity deviates from 1, the concavity in the nucleus increases. It is calculated following the equation, $\eta_{\sigma} = \frac{\alpha}{\mathscr{A}}$, with $\mathscr{A}$ representing the area of the convex hull that best encloses the nucleus boundary. The results are shown in the Supplementary Figure S2(f) and indicate that most of the nuclei have values close to 1.

The averages for each shape descriptor are shown in the Supplementary Table S3 and the plots in the Supplementary Figure S3. The standard deviations range is the shaded region. The standard error of the mean is shown as a black line around the data point. Data points are presented with different symbols according to the day at which the image was taken (see legend panel f).

The behaviour of the shape descriptor, although useful at the colony level for the detection of morphological changes between pluripotent and differentiated colonies, do not show a significant change at the nucleus level. The aspect ratio of the nuclei is ~ 1.5 in all cases, see Supplementary Figure S3(a).

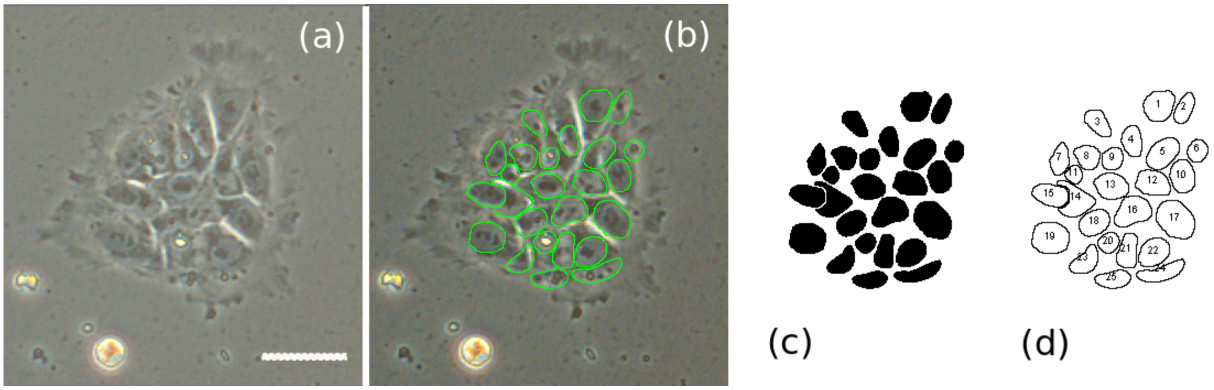

Supplementary Figure S1. (a) Example of the cell nuclei detection in a hESC colony. (b) The tracing was performed by outlining the nuclei. (c) This information was extracted and transformed in a binary file. (d) Using the plugin "Analyze Particles" in ImageJ [39] we detected each particle and measured the properties mentioned in the text. Scale bar 50 μm.

Edge detection

To detect the colony edges from the phase contrast images we use the canny Deriche algorithm included in ImageJ [39] to aid in the identification of the border as shown in Supplementary Figure S8(a) and (b). To obtain the boundary we traced manually the borders obtained in (b).

Colony reconstruction

To measure the correlation between the cell nucleus size and its position within the colony we reconstructed the colony shown in Figure 2 using the Voronoi diagram (VD) bounded by the colony's boundaries. In the Supplementary Figure S6(a) we show the reconstructed VD for the colony in Figure 2. Using the centroid position for each cell we calculated their (closest) distance Λ from the boundary and plotted this as a function of the cell nucleus area α, see Supplementary Figure S6(b). Our results indicate that the cell sizes are not correlated to their position within the colony, with a correlation coefficient of $R = 0.091$.

Structural analysis through the radial distribution function

Radial distribution functions (RDF) are tools used widely in crystallography and soft condensed matter physics to characterise the structure of different materials [54]. The RDF, denoted with $g(r)$, determines the number of particles in a spherical shell of radius r and thickness dr, i.e., $ng(r)4\pi r^2 dr$. In other words, it describes the variation of the local cell density within a distance r as viewed from the centring cell, relative to its bulk value. For ordered materials, such as crystals, the radial distribution function show an oscillating behaviour, where the peaks in $g(r)$ are interpreted as the average inter-particle distances. The information contained in the RDF is a spatial average and has its limitations when the system is not isotropic.

Supplementary Figure S7 show results for the RDF for two colonies of different sizes with areas $0.690\,\mathrm{mm}^2$ and $1.131\,\mathrm{mm}^2$, respectively. For both cases, the first peak is the best-defined one and corresponds to the distribution of the distance between the first nearest neighbours $\ell_1 \sim 18.56\,\mu$m and $\ell_1 \sim 18.02\,\mu$m respectively. The position of the second peak, gives the average distance or coordination, between second neighbours ℓ_2. We conclude that the colonies show a short-range order and the nearest coordination shells are visible in both cases. But, as we increase r to account for the second nearest neighbours the second peak is washed out and broader than the first due to missing long-range order. The largest colony shows a second peak centred around $\ell_2 \sim 40.2\,\mu$m. Interestingly, the RDF shows that the cells in the largest colony ($A = 1.131\,\mathrm{mm}^2$) are more packed since the blue curve crosses the horizontal dotted line before the green curve. The results are shown in Supplementary Figure S7 and indicate that $g(r)$ is similar to the ones obtained for amorphous materials, therefore the structure of the material becomes blurred as the radius r increases.

In biology, calculations of $g(r)$ have been performed to study protein organisation, aggregation of particles on cell membranes, [55, 56]. This tool has not been used previously in the literature to characterise dense aggregates of cells.

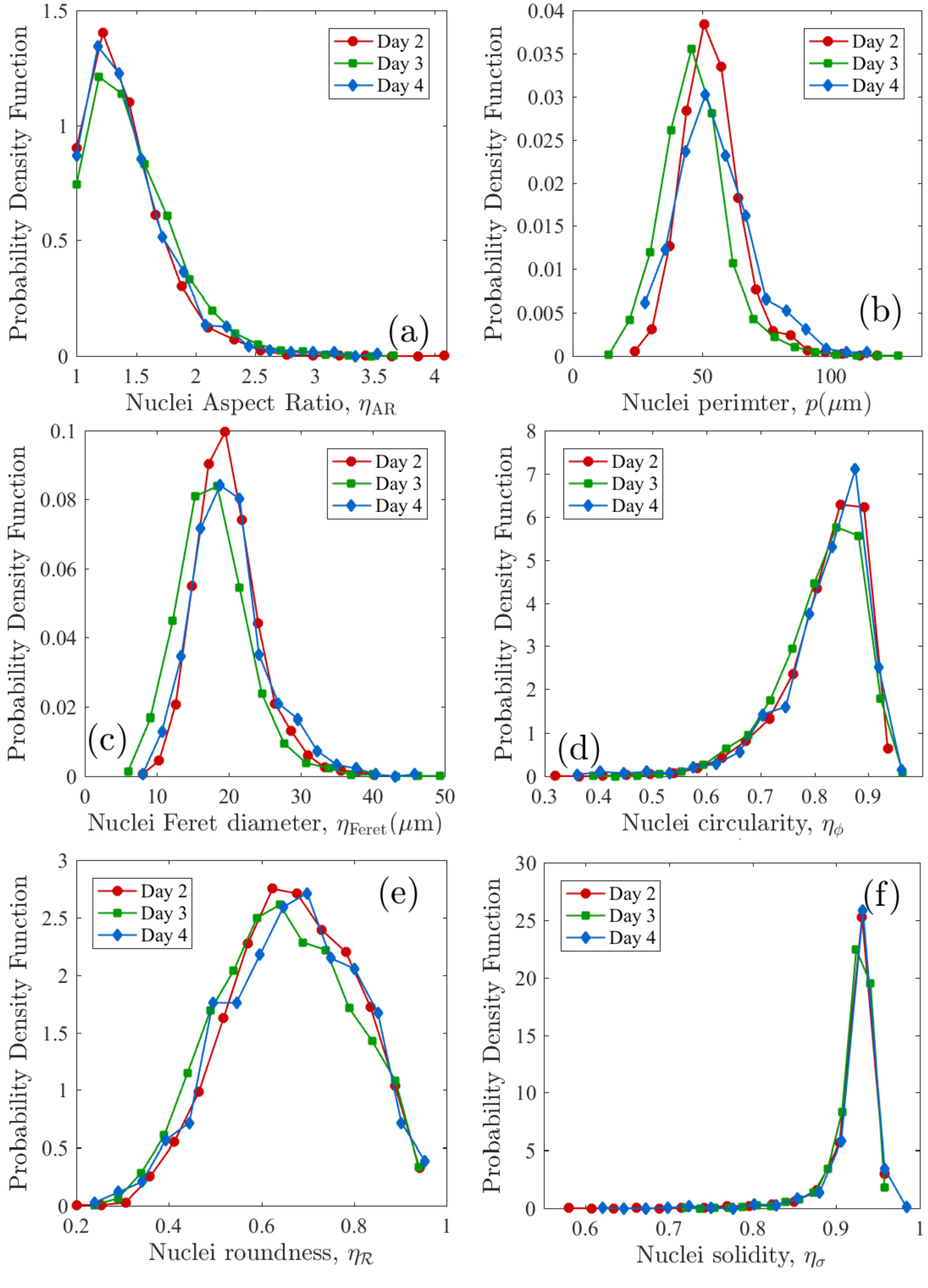

Supplementary Figure S2. Probability density functions (PDF) for the nuclei shape descriptors: (a) aspect ratio η_{AR}, (b) perimeter p, (c) Feret's diameter η_{Feret}, (d) circularity η_{ϕ}, (e) roundness $\eta_{\mathscr{R}}$ and (f) solidity η_{σ}.

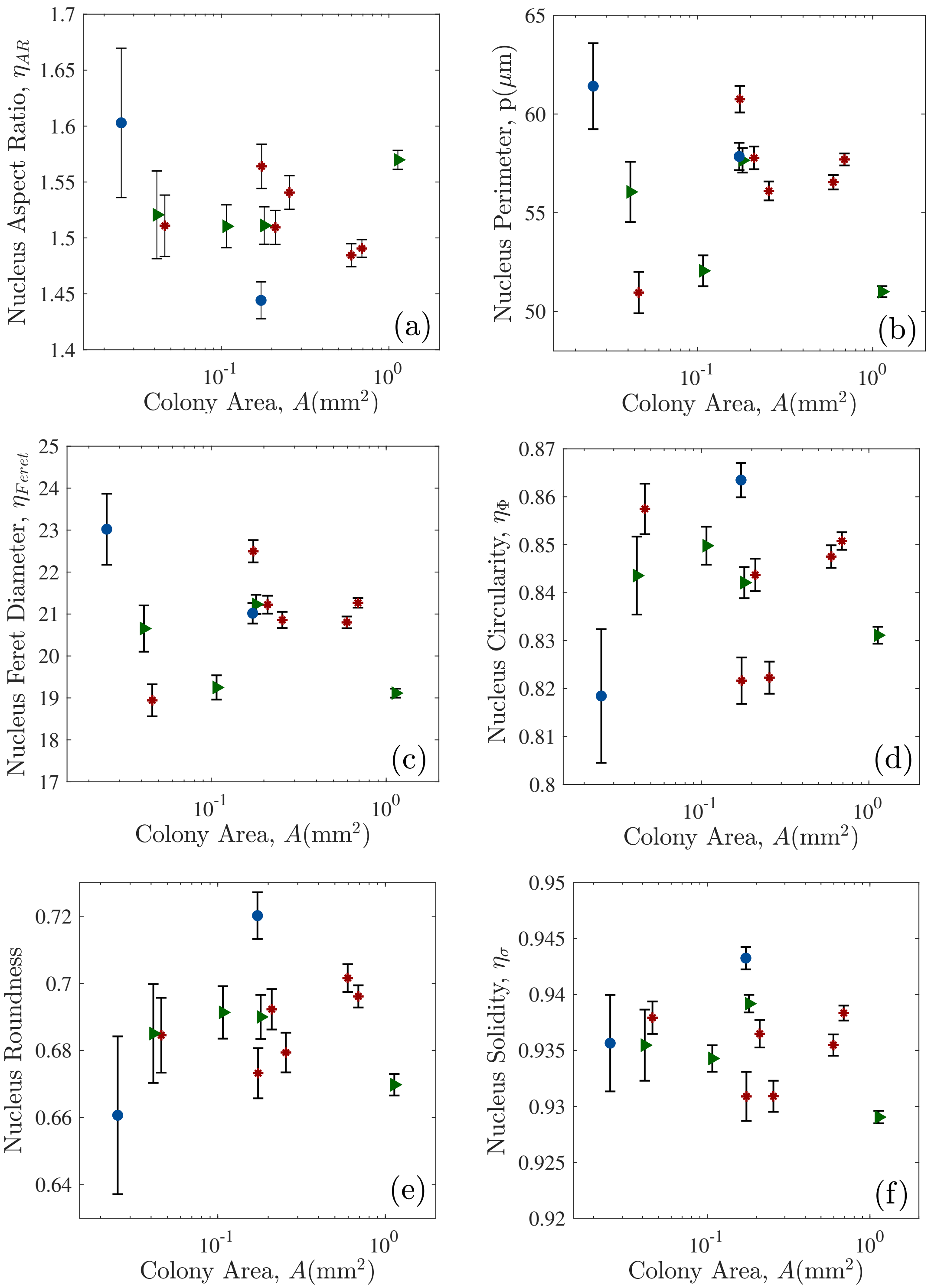

Supplementary Figure S3. Mean values of the shape descriptors obtained for the nuclei of the cells. The results are shown according to the sampling day, see the legend in f. The average value for the nucleus aspect ratio is $\eta_{\mathrm{AR}} \sim 1.5$. The nuclei perimeter p is between the range $[50, 60]$. The Feret's diameter η_{Feret} is highest for the colonies with $A \approx 0.025$ mm^2, that contain differentiated cells at day 4, (blue • to the left). The perimeter p decreases for larger colonies. A similar trend is shown in c for the Feret's diameter. The remaining shape descriptors corroborate that, in general, most of the nuclei are round and circular in shape, without irregular borders. The error bars shown are the standard errors of the mean.

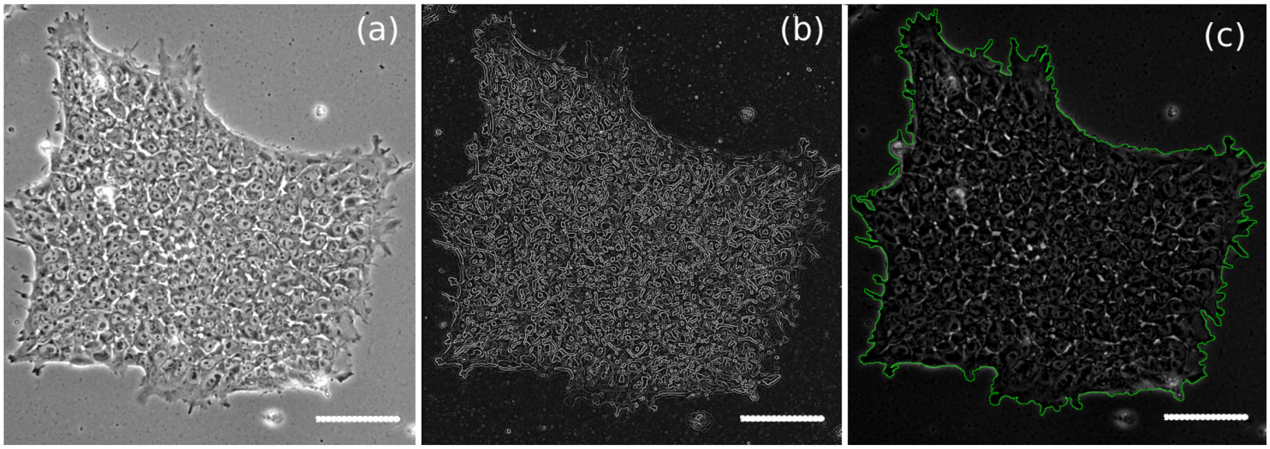

Supplementary Figure S4. (a) Human ESCs colony at day 3 processed with (b) the canny Deriche algorithm to obtain (c) the boundary.

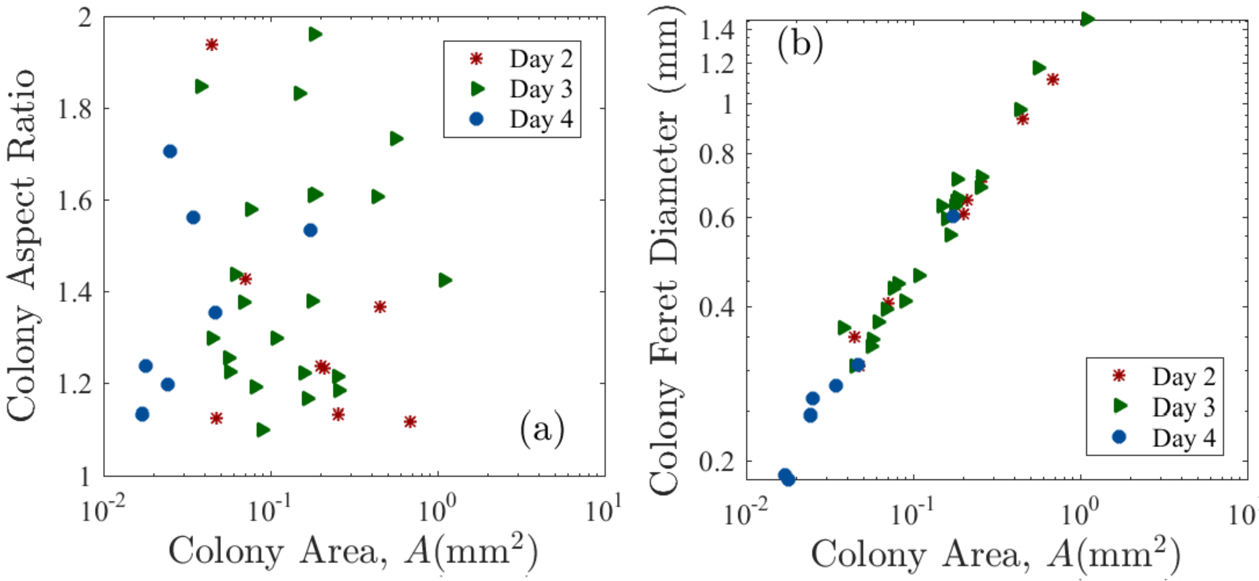

Supplementary Figure S5. Numerical values of the (a) aspect ratio and (b) the Feret's diameter for the colonies shown in the Supplementary Table S4.

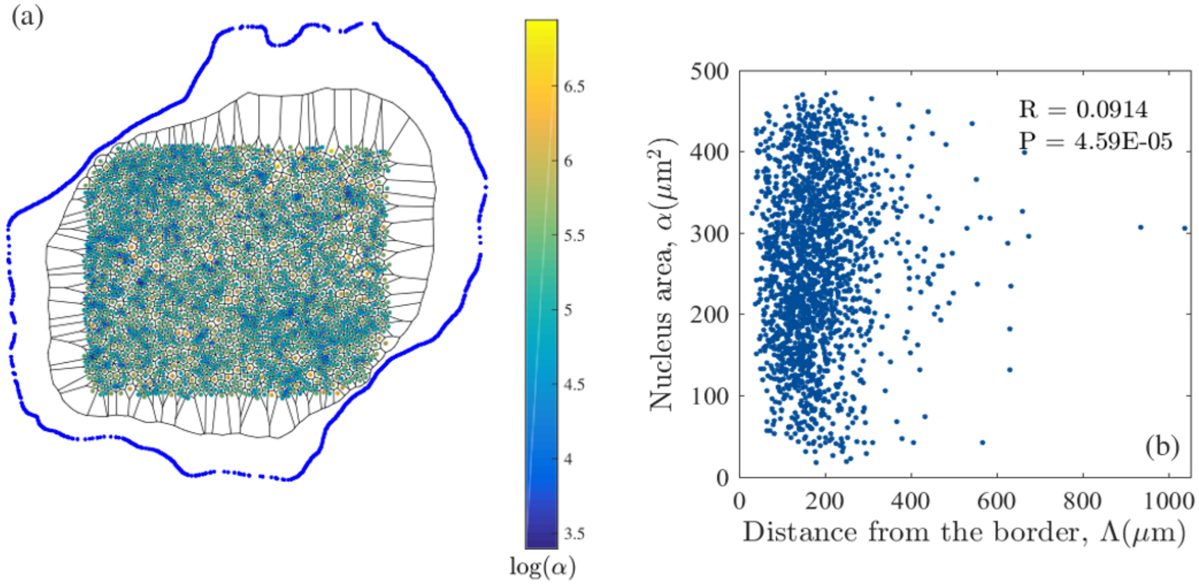

Supplementary Figure S6. (a) Voronoi tessellation constructed for the colony shown in Figure 2, with an area $A = 1.131\,\mathrm{mm}^2$ and $N_c = 1982$. The border of the colony is shown with a blue dotted line. (b) Cell nucleus area (α) as a function of their distance $\Lambda\,(\mu\mathrm{m})$ from the colony edge. The correlation coefficient is $R = 0.091$ and indicates a null dependence between both variables.

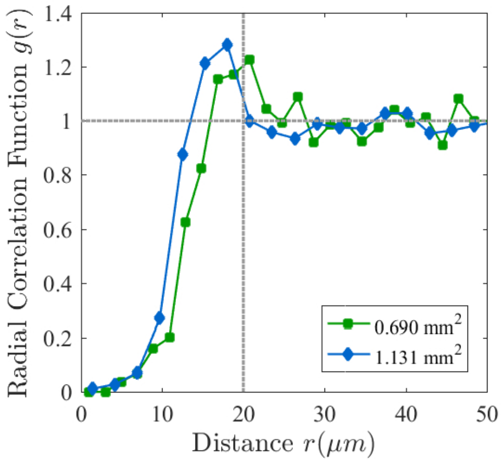

Supplementary Figure S7. Radial distribution functions $g(r)$ for the two largest colonies analysed. The first peak, associated with the separation of the first nearest neighbours indicates a location of $< 20\,\mu\mathrm{m}$, consistent with the results presented in Figure 4 obtained through the Voronoi diagram.

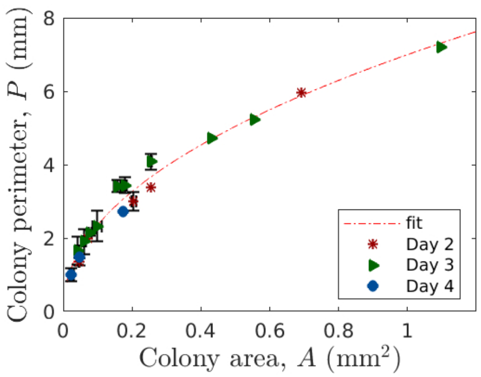

Supplementary Figure S8. Colony perimeter P as a function of the colony area A. These datapoints were obtained by applying the canny Deriche algorithm to the samples. The red dashed-dotted line shows the best fit to a power function with a scaling factor $\kappa = 7.207$ and exponent $\gamma = 0.47$, (R^2 = 0.963).

Supplementary Table S2. Measurements obtained for the nuclei morphology and cellular parametric characteristics in hESC colonies. The total number of cells in the colonies N_c, the mean cell nucleus area $\langle\alpha\rangle$, the mean number of nearest neighbours $\langle N_n\rangle$ and the mean intracellular distance $\langle\ell_n\rangle$, alongside the standard error of the mean and the standard deviations (within parenthesis) for the measurements.

A (mm^2)	N_c	$\langle\alpha\rangle$, μm^2	$\langle N_n\rangle$	$\langle\ell_n\rangle$, μm	Day
$0.025 \pm 0.007^{\dagger}$	39 ± 10	269 ± 18 (111)	4.81 ± 0.22 (1.40)	22.16 ± 0.53 (3.30)	4
0.041 ± 0.005	71 ± 34	212 ± 12 (105)	5.08 ± 0.17 (1.41)	20.48 ± 0.37 (3.11)	3
0.046 ± 0.002	115 ± 1.4	185 ± 8 (82)	5.20 ± 0.13 (1.44)	20.31 ± 0.35 (3.73)	2
0.107	305	206 ± 5 (96)	5.57 ± 0.07 (1.23)	18.43 ± 0.15 (2.61)	3
0.173	363	239 ± 6 (109)	5.61 ± 0.06 (1.15)	21.60 ± 0.16 (3.13)	4
0.174	375	248 ± 5 (100)	5.63 ± 0.06 (1.16)	21.46 ± 0.16 (3.10)	2
0.180	409	230 ± 5 (104)	5.60 ± 0.05 (1.26)	21.29 ± 0.15 (3.06)	3
0.210	514	232 ± 4 (99)	5.67 ± 0.05 (1.14)	20.85 ± 0.13 (3.06)	2
0.255	543	226 ± 3 (80)	5.62 ± 0.04 (1.28)	20.67 ± 0.13 (3.14)	2
0.596	1026	222 ± 3 (92)	5.76 ± 0.03 (1.12)	20.37 ± 0.10 (3.15)	2
0.690	1489	211 ± 2 (91)	5.84 ± 0.03 (1.03)	20.13 ± 0.08 (3.08)	2
$1.131^{\ddagger}$	1982	179 ± 2 (87)	5.91 ± 0.02 (0.96)	18.56 ± 0.07 (3.11)	3

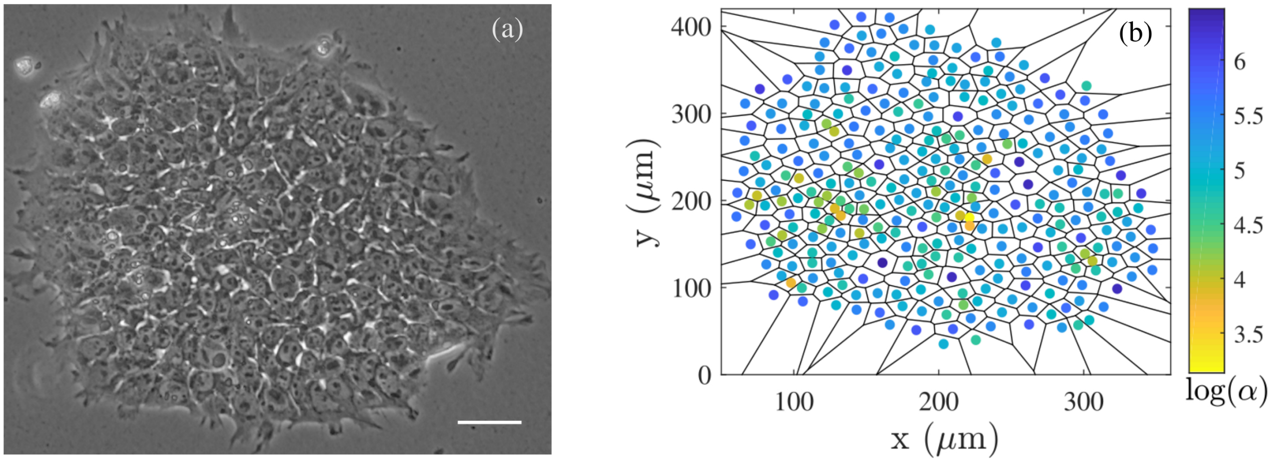

Supplementary Figure S9. (a) Colony with an area $A = 0.107\,\mathrm{mm}^2$ and $N_c = 305$ cells imaged at day 3. The mean nucleus area is $\langle\alpha\rangle = 206 \pm 96\,\mu\mathrm{m}^2$ and the mean intercellular distance is $\langle\ell_n\rangle = 18.43 \pm 2.61\,\mu\mathrm{m}$. Bar $50\,\mu\mathrm{m}$. (b) Voronoi diagram of the colony shown in (a). The colour bar indicates the logarithm of the cell area (α). Patches of small cells (green and yellow) are visible in several regions of the colony.

Supplementary Table S3. Shape descriptors obtained for the nuclei features in hESC colonies: aspect ratio η_{AR}, perimeter p, Feret's diameter η_{Feret}, circularity η_{Φ}, roundness $\eta_{\mathscr{R}}$ and solidity η_{σ}. We show in the first column the area of the colony and in the last the sampling day. The standard error is shown on each measurement alongside the standard deviation withing parenthesis.

A (mm^2)	η_{AR}	p	η_{Feret}	η_{ϕ}	$\eta_{\mathscr{R}}$	η_{σ}	Day
0.025 ± 0.007†	1.60 ± 0.07 (0.42)	61.41 ± 2.18 (13.66)	23.02 ± 0.85 (5.30)	0.82 ± 0.014 (0.09)	0.66 ± 0.023 (0.15)	0.95 ± 0.004 (0.03)	4
0.041 ± 0.005	1.52 ± 0.04 (0.33)	56.06 ± 1.52 (12.89)	20.65 ± 0.55 (4.66)	0.84 ± 0.008 (0.07)	0.69 ± 0.015 (0.12)	0.93 ± 0.003 (0.03)	3
0.046 ± 0.002	1.51 ± 0.03 (0.29)	50.96 ± 1.05 (11.23)	18.94 ± 0.38 (4.09)	0.86 ± 0.005 (0.06)	0.68 ± 0.011 (0.12)	0.94 ± 0.002 (0.02)	2
0.107	1.51 ± 0.02 (0.33)	52.06 ± 0.78 (13.64)	19.25 ± 0.29 (5.06)	0.85 ± 0.004 (0.07)	0.69 ± 0.008 (0.14)	0.93 ± 0.001 (0.02)	3
0.173	1.44 ± 0.02 (0.31)	57.85 ± 0.69 (13.19)	21.02 ± 0.24 (4.67)	0.86 ± 0.004 (0.07)	0.72 ± 0.007 (0.13)	0.94 ± 0.001 (0.02)	4
0.174	1.56 ± 0.02 (0.38)	60.75 ± 0.67 (13.05)	22.50 ± 0.27 (5.14)	0.82 ± 0.005 (0.09)	0.67 ± 0.008 (0.14)	0.93 ± 0.001 (0.04)	2
0.180	1.51 ± 0.02 (0.34)	57.66 ± 0.62 (12.56)	21.23 ± 0.23 (4.62)	0.84 ± 0.003 (0.07)	0.69 ± 0.007 (0.13)	0.94 ± 0.002 (0.01)	3
0.210	1.51 ± 0.02 (0.35)	57.78 ± 0.57 (13.03)	21.22 ± 0.21 (4.83)	0.84 ± 0.003 (0.07)	0.69 ± 0.006 (0.14)	0.94 ± 0.001 (0.03)	2
0.255	1.54 ± 0.01 (0.35)	56.11 ± 0.50 (11.16)	20.86 ± 0.19 (4.50)	0.82 ± 0.003 (0.08)	0.68 ± 0.006 (0.14)	0.93 ± 0.001 (0.03)	2
0.596	1.48 ± 0.01 (0.33)	56.54 ± 0.36 (11.65)	20.80 ± 0.14 (4.48)	0.85 ± 0.002 (0.07)	0.70 ± 0.004 (0.13)	0.94 ± 0.001 (0.03)	2
0.690	1.49 ± 0.01 (0.30)	57.70 ± 0.30 (11.69)	21.27 ± 0.11 (4.40)	0.85 ± 0.002 (0.07)	0.70 ± 0.003 (0.13)	0.94 ± 0.001 (0.03)	2
1.131‡	1.57 ± 0.01 (0.38)	51.00 ± 0.28 (12.37)	19.11 ± 0.11 (4.77)	0.83 ± 0.002 (0.08)	0.67 ± 0.003 (0.14)	0.93 ± 0.001 (0.02)	3

† For the smallest colonies we obtained statistics from several samples, grouping them according to imaging day.
‡ Only a portion of the colony was analysed.

Supplementary Table S4. Datasets of the morphological and parametric characteristics for hESC colonies. We show the colony area A, number of cells N_c, perimeter P, circularity Φ, Feret diameter, minimum Feret diameter, aspect ratio L, roundness $\mathscr{R}$, solidity Σ and day of imaging. The colony identifier also indicates the zoom at which the image was taken in the last two characters: $\times 5$ or $\times 10$.

Tag	Identifier	A(mm^2)	N_c	P (μm)	Φ	Feret	MinFeret	L	$\mathscr{R}$	Σ	Day
1	DAY2_4x5	0.691	1489	5.960	0.245	1115.1	953.2	1.117	0.895	0.874	2
2	DAY2_6x10	0.456	1026	2.747	0.759	931.6	691.4	1.365	0.733	0.962	2
3	DAY2_7x10	0.209	514	3.180	0.260	644.8	513.1	1.233	0.811	0.840	2
4	DAY2_8Ax10	0.047	114	1.428	0.292	305.6	252.3	1.125	0.889	0.810	2
5	DAY2_8Bx10	0.045	116	1.283	0.341	348.5	190.7	1.938	0.516	0.845	2
6	DAY2_9x10	0.071	206	1.979	0.228	405.0	275.3	1.428	0.700	0.827	2
7	DAY2_10x10	0.254	543	3.390	0.278	702.4	573.3	1.132	0.883	0.883	2
8	DAY2_11x10	0.199	375	2.825	0.314	607.3	481.8	1.238	0.808	0.872	2
9	DAY3_1x10	0.164	457	3.249	0.196	553.2	452.5	1.165	0.858	0.842	3
10	DAY3_2X10	0.056	151	1.770	0.226	344.5	279.6	1.224	0.817	0.816	3
11	DAY3_3x10	0.255	862	3.930	0.208	716.1	555.3	1.183	0.846	0.845	3
12	DAY3_4x10	0.107	305	2.616	0.197	460.5	356.8	1.298	0.770	0.873	3
13	DAY3_5x10	0.080	198	2.187	0.211	444.4	341.4	1.192	0.839	0.763	3
14	DAY3_6x5	1.097	-	7.205	0.263	1460.6	1040.9	1.420	0.703	0.888	3
15	DAY3_7x10	0.252	703	4.230	0.177	685.4	529.9	1.213	0.824	0.848	3
16	DAY3_10x10A	0.056	83	1.535	0.299	334.2	275.7	1.255	0.797	0.839	3
17	DAY3_10x10B	0.038	74	1.922	0.129	363.6	215.7	1.848	0.541	0.651	3
18	DAY3_11x10	0.088	229	2.023	0.270	410.0	341.6	1.100	0.909	0.837	3
19	DAY3_12x10	0.069	166	1.983	0.219	394.8	289.6	1.376	0.727	0.803	3
20	DAY3_14x10	0.182	440	3.664	0.171	710.0	391.8	1.960	0.510	0.844	3
21	DAY3_16x10	0.159	359	3.410	0.172	594.0	462.6	1.223	0.818	0.778	3
22	DAY3_17x5	0.430	-	4.718	0.243	974.8	614.9	1.607	0.622	0.876	3
23	DAY3_18x10	0.175	-	3.320	0.200	629.9	461.7	1.379	0.725	0.830	3
24	DAY3_19x10	0.076	-	2.095	0.217	433.9	292.2	1.579	0.633	0.775	3
25	DAY3_20x10	0.180	409	3.222	0.218	643.6	439.3	1.609	0.622	0.833	3
26	DAY3_21x10	0.147	-	3.593	0.143	629.5	380.3	1.831	0.546	0.792	3
27	DAY3_23x5	0.555	-	5.232	0.255	1172.1	746.7	1.734	0.577	0.857	3
28	DAY3_24x10	0.061	-	2.341	0.140	372.2	291.9	1.438	0.695	0.764	3
29	DAY3_25x10	0.045	69	1.390	0.291	305.2	231.7	1.297	0.771	0.858	3
30	DAY3_26x10	0.184	-	3.560	0.183	654.2	434.8	1.610	0.621	0.839	3
31	DAY4_18x10	0.017	25	0.871	0.281	187.7	157.7	1.132	0.883	0.825	4
32	DAY4_3x10	0.173	363	2.734	0.290	600.6	413.2	1.534	0.652	0.862	4
33	DAY4_7x10	0.047	86	1.474	0.270	307.1	224.6	1.354	0.739	0.844	4
34	DAY4_8x10	0.024	40	1.237	0.200	245.1	173.2	1.197	0.836	0.766	4
35	DAY4_9x10	0.035	46	1.083	0.372	280.2	188.7	1.562	0.640	0.869	4
36	DAY4_11x10	0.025	46	1.064	0.279	263.2	162.8	1.706	0.586	0.765	4
37	DAY4_21x10	0.018	52	0.791	0.359	184.1	157.7	1.236	0.809	0.827	4

– Cells were not counted, only features analysed.

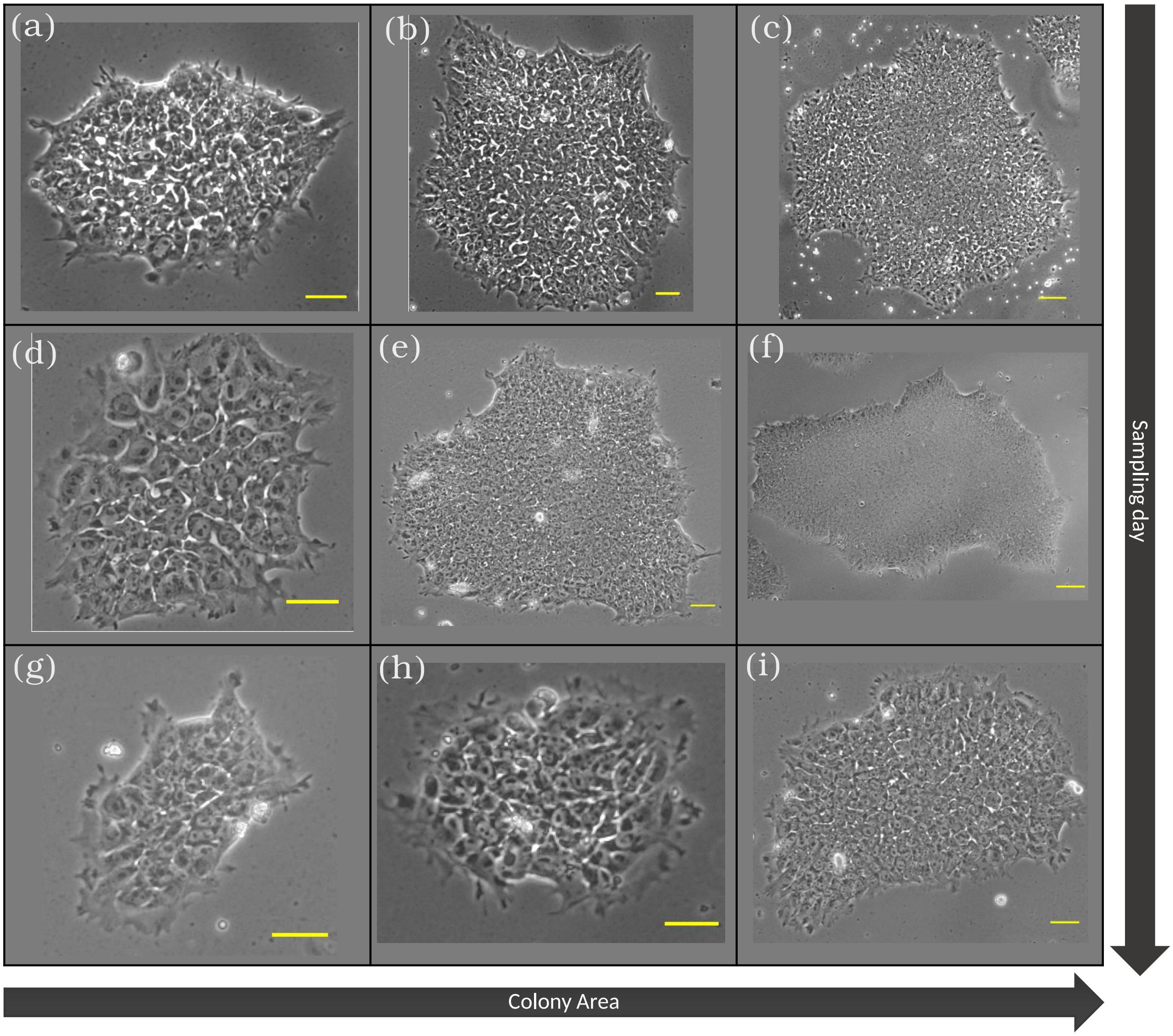

Supplementary Figure S10. (a-c) Phase-contrast images of hESC colonies analysed at day 2 with areas $A = 0.071$, 0.254 and 0.691mm^2, respectively. (e-f) Day 3 colonies with areas $A = 0.056$, 0.255 and 0.555mm^2 and (g-i) day 4 colonies with $A = 0.025$, 0.047 and 0.173mm^2 Bars 50μm in a, b, d, e, g, h, i and 100μm in c and f.

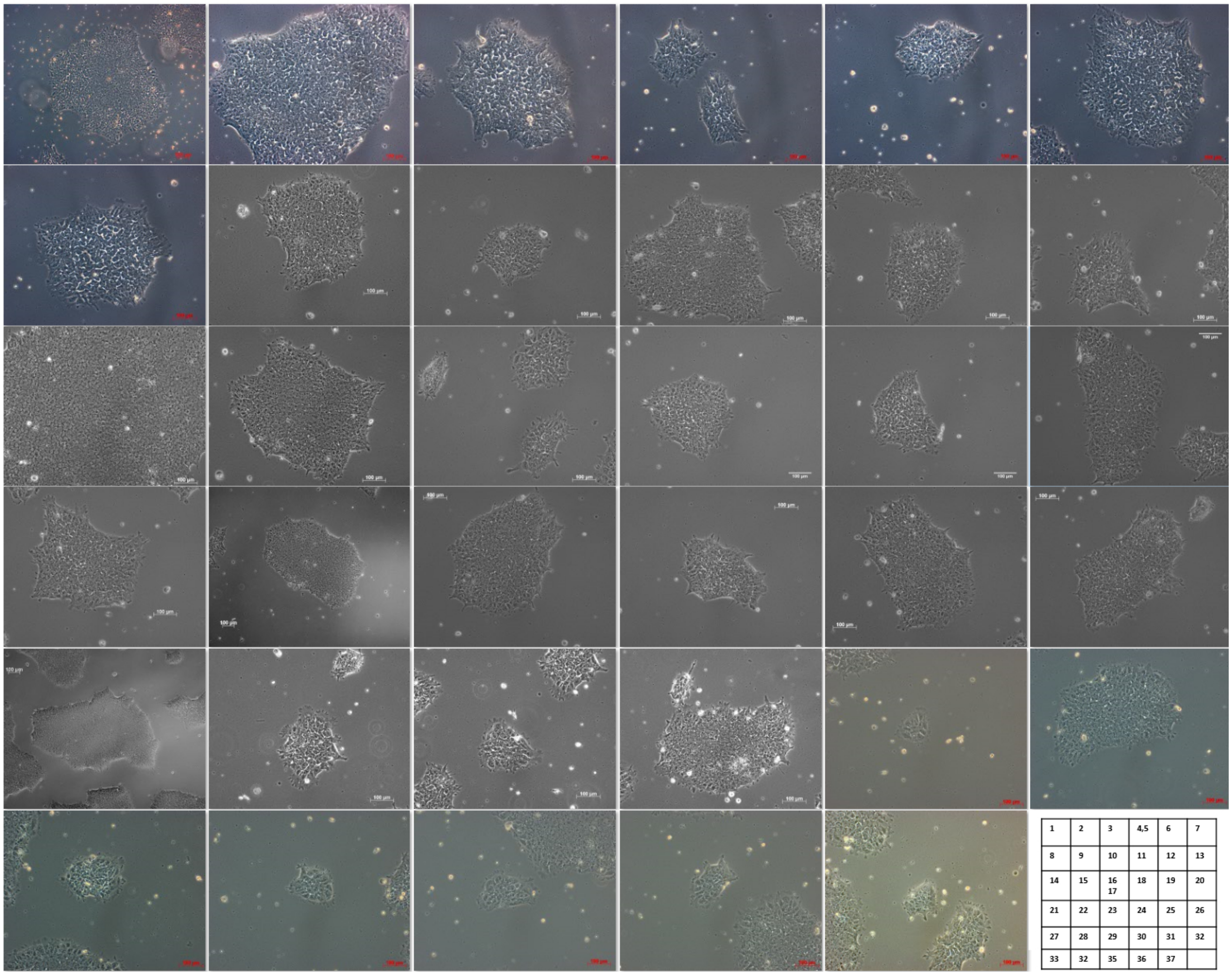

1	2	3	4,5	6	7
8	9	10	11	12	13
14	15	16<br>17	18	19	20
21	22	23	24	25	26
27	28	29	30	31	32
33	32	35	36	37	

Supplementary Figure S11. Gallery of colony images used for the morphological analysis. The images are labelled in the grid at the bottom-right following the tags shown in Supplementary Table S2. For example: In the grid 1, we have the colony with tag 1 in Supplementary Table S2, which corresponds to the identifier DAY2_4x5. This indicates that the colony was imaged at day 2 using a magnification 5×. The grids with two tags, i.e., 4,5 and 16, 17, show two colonies analysed in the same image. Scale bar 100 μm.
